# Comparative Transcriptomics Reveals Differential Gene Expression Related to *Colletotrichum gloeosporioides* Resistance in the Octoploid Strawberry

**DOI:** 10.3389/fpls.2017.00779

**Published:** 2017-05-15

**Authors:** Feng Wang, Feng Zhang, Mengmeng Chen, Zhiheng Liu, Zhihong Zhang, Junfan Fu, Yue Ma

**Affiliations:** ^1^College of Plant Protection, Shenyang Agricultural UniversityShenyang, China; ^2^College of Horticulture, Shenyang Agricultural UniversityShenyang, China; ^3^Institute of Crop Science, Chinese Academy of Agricultural SciencesBeijing, China

**Keywords:** strawberry, *Colletotrichum gloeosporioides*, transcriptome, resistance gene, quantification of gene expression

## Abstract

The strawberry is an important fruit worldwide; however, the development of the strawberry industry is limited by fungal disease. Anthracnose is caused by the pathogen *Colletotrichum gloeosporioides* and leads to large-scale losses in strawberry quality and production. However, the transcriptional response of strawberry to infection with *C. gloeosporioides* is poorly understood. In the present study, the strawberry leaf transcriptome of the ‘Yanli’ and ‘Benihoppe’ cultivars were deep sequenced via an RNA-seq analysis to study *C. gloeosporioides* resistance in strawberry. Among the sequences, differentially expressed genes were annotated with Gene Ontology terms and subjected to pathway enrichment analysis. Significant categories included defense, plant–pathogen interactions and flavonoid biosynthesis were identified. The comprehensive transcriptome data set provides molecular insight into *C. gloeosporioides* resistance genes in resistant and susceptible strawberry cultivars. Our findings can enhance breeding efforts in strawberry.

## Introduction

Cultivated strawberry (*Fragaria* × *ananassa* Duch) is an important fruit worldwide and is popular with consumers because of its flavor, fragrance and nutritional value. Anthracnose is one of the most destructive diseases in strawberry cultivation areas. In strawberry, anthracnose is mainly caused by three fungal pathogens: *Colletotrichum gloeosporioides, Colletotrichum fragariae*, and *Colletotrichum acutatum* (Peres et al., 2005). *C. gloeosporioides* typically causes petiole and stolon lesions and crown rot in strawberry and can also produce symptoms in the fruit (McInnes et al., 1992). With the expansion of strawberry planting areas in China, anthracnose is causing increasingly significant harm to China’s strawberry industry. The strawberry greenhouse planting area is gradually expanding in Northern China, and the spread of strawberry anthracnose disease is significantly impacting the development of the strawberry industry in this region.

In strawberry, significant efforts have been devoted to revealing the molecular components associated with anthracnose resistance. Subtractive hybridization (Casado-Díaz et al., 2006), microarray (Guidarelli et al., 2011), and proteomics (Fang et al., 2012) have been used to identify the genes associated with anthracnose infection. Li et al. (2013) identified a set of NB-LRR genes that display ecotype-specific responses to *C. gloeosporioides* inoculation via the genome-wide isolation and characterization of NB-LRR genes in woodland strawberry. Zhang et al. (2016) found that NB-LRR transcripts accumulate in response to SA pretreatment and that SA directly inhibits the germination of *C. gloeosporioides*. Amil-Ruiz et al. (2016) revealed that specific aspects of SA- and JA-dependent signaling pathways are activated in strawberry upon interaction with *C. acutatum*. However, the components of the strawberry defense network remain largely unknown or poorly understood, and the exact mechanisms have not been identified.

In this study, we performed disease assessment of the four main strawberry cultivars (‘Yanli,’ ‘Toyonoka,’ ‘Sachinoka’ and ‘Benihoppe’) in northern China following infection with *C. gloeosporioides*. We compared the dynamic changes in gene expression during the process of infection with *C. gloeosporioides*. We generated a comparative transcriptome between ‘Yanli’ and ‘Benihoppe,’ with three different libraries for each cultivar. These data deepen our understanding of the complex genetic and molecular mechanisms of strawberry defense.

## Materials and Methods

### Plant Materials

Plant tissue-cultured material from strawberry cultivars ‘Yanli,’ ‘Toyonoka,’ ‘Sachinoka’ and ‘Benihoppe’ was grown in a greenhouse at the Fruit Molecular Biology Laboratory of Shenyang Agricultural University. The experiment was organized into two groups, with 20 strains per variety in each group. The plants were selected for uniform size and color and an absence of visual defects. The plants were managed under identical conditions. Collected leaves were frozen immediately in liquid nitrogen and stored at -80°C until further use.

### *Colletotrichum gloeosporioides* Culture and Infection

The pathogenic fungi used for the experiments were grown in shaking Potato Dextrose (PD) cultures for 7–10 days at a culture temperature of 25°C and then were filtered through four layers of gauze into a diluent. The accession number of the internal transcribed spacer (ITS) sequence of the *C. gloeosporioides* strain is KT224457. After incubation, the suspension population was counted using a bacterial counting chamber and adjusted to 10^6^ spores ml^-1^. Then, the conidial suspension was transferred to a watering can, and the plant leaves and stems were sprayed to cover their surfaces with a uniform layer of the suspension. The inoculated leaves were subsequently incubated at 25°C for disease development. The results were recorded after 0, 24, 72, 96, and 120 h.

### Disease Assessment

External symptoms of disease were indicated by any sign of disease spots on the plants. The disease incidence and disease index (DI) on each leaf were observed and recorded after incubation. When the spot zone beyond the wounded site on a leaf was greater than 1 mm wide, the site was scored as infected. The disease incidence was expressed as the percentage (%) of infected leaves among the total leaves. The disease index of each leaf was rated according to the percentage of observed spots on a scale of 0–5: (0) no disease spots; (1) <5% disease spot area; (2) 5–10% disease spot area; (3) 10–15% disease spot area; (4) 15–20% disease spot area; and (5) >20% disease spot area.

(1) DI=Σ(number of diseased plants in this index×disease index)total number of leaves×maximum disease index×100

### RNA Isolation and Quality Assessment

Total RNA was isolated from the plant leaves using the modified CTAB method as described by Chang et al. (2007). The RNA samples were treated with DNase (TaKaRa, Japan) for 4 h. The integrity of the RNA samples was examined using an Agilent 2100 Bioanalyzer (Agilent Technologies, Palo Alto, CA, United States).

### cDNA Library Preparation and Illumina Sequencing

For the RNA-seq experiments, the strawberry cultivars ‘Yanli’ (resistant) and ‘Benihoppe’ (susceptible) were used. The samples included 0-h-untreated ‘Yanli’ (R1), 72-h-untreated ‘Yanli’ (R2) 72-h-infected (with *C. gloeosporioides*) ‘Yanli’ (R3), 0-h-untreated ‘Benihoppe’ (S1), 72-h-untreated ‘Benihoppe’ (S2) and 72-h-infected ‘Benihoppe’ (S3). Leaves were randomly collected for each treatment. A total of six pools were submitted to the OEbiotech Company (Shanghai, China) for cDNA library preparation and sequencing. The paired-end library preparation and sequencing were performed following standard Illumina methods using a DNA sample kit (#FC-102-1002, Illumina). The cDNA library was sequenced on the HiSeq^TM^ 2500 Illumina sequencing platform.

### Mapping Reads to the Reference Genome

Clean reads were preprocessed by removing the adaptor sequences and discarding the empty and low-quality sequences. The reads were then mapped to the octoploid strawberry genome FAN_r1.1^1^ (Hirakawa et al., 2013) using Tophat with the default parameters.

### Functional Annotation

We annotated genes based on a set of sequential BLAST searches (Altschul et al., 1997) to identify the most descriptive annotation for each sequence. The genes were compared with sequences in the National Center for Biotechnology Information (NCBI) non-redundant (Nr) protein databases^2^ and the Swiss-Prot protein database^3^. The genes were also queried against the Plant Resistance Gene database^4^.

### Digital Gene Expression Analysis

Gene expression levels in the RNA-seq analysis were measured as reads per kilobase of exon model per million mapped reads (RPKM). DESeq software^5^ was used to identify differentially expressed genes (DEGs) via pairwise comparisons, and the results of all statistical tests were adjusted for multiple testing with the Benjamini—Hochberg false discovery rate (FDR < 0.01). Sequences were deemed to be significantly differentially expressed if the adjusted *P*-value obtained by this method was <0.05 and at least a two-fold change (>1 or <-1 in log 2 ratio value) in the RPKM was observed between the two libraries.

### GO and KEGG Pathway Enrichment Analysis

The functions of the DEGs were characterized using Gene Ontology (GO) terms with the agriGO database (Du et al., 2010). The second method used for DEG characterization was based on a Kyoto Encyclopedia of Genes and Genomes (KEGG) pathway enrichment analysis. Pathways with *Q*-values < 0.05 were considered significantly enriched in DEGs. The *Q*-value was used to determine the *P*-value threshold (Benjamini and Hochberg, 1995).

### qRT-PCR Assay

First-strand cDNA was synthesized from one microgram of total RNA using the PrimeScript RT reagent kit with gDNA Eraser (TaKaRa, Japan) according to the manufacturer’s instructions.

To determine the expression of DEGs, quantitative reverse transcription PCR (qRT-PCR) was performed on the iQ5 Real-Time PCR Detection System (Bio-Rad, United States) using SYBR Premix Ex Taq (TaKaRa, Japan). The cycling conditions were as follows: 40 cycles of denaturation at 95°C for 30 s, annealing at 58°C for 45 s, and extension at 72°C for 30 s. A dissociation curve was generated at the end of each PCR cycle to verify that a single product had been amplified using software provided with the iQ5 Real-Time PCR Detection System. Each gene was analyzed in triplicate, and then the average threshold cycle (CT) was calculated per sample. All of the data were normalized to the level of the Fv26S internal transcript control. Relative fold changes in gene expression were calculated using the comparative Ct method (2^-ΔΔCT^) (Liu C. et al., 2012; Liu T. et al., 2012). Primers used in the PCR analysis are listed in **Supplementary Table S3**.

### Statistical Analysis

The data were statistically analyzed using DPS 7.05 software. After conducting an analysis of variance (ANOVA), Duncan’s test was used to identify significant differences between mean values.

## Results

### Differential Responses of Strawberry Cultivars to *C. gloeosporioides*

**FIGURE 1:**
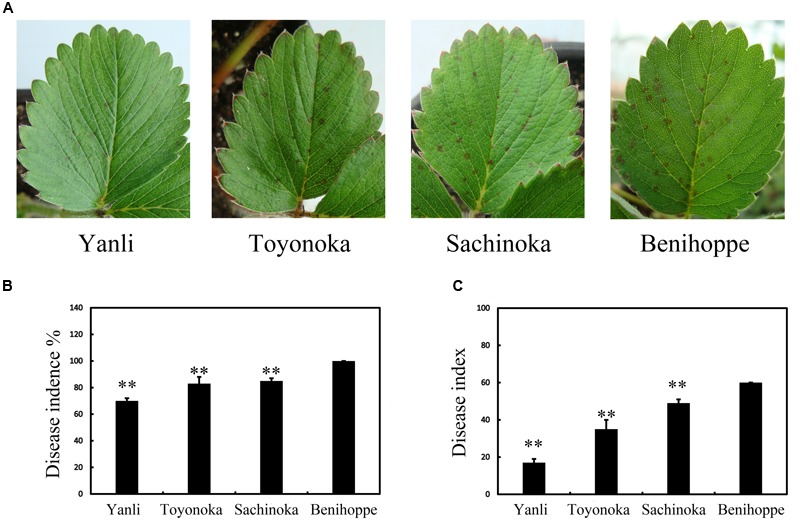
**Disease symptoms on the leaves of strawberry after *C. gloeosporioides* infection. (A)** Disease symptoms on ‘Yanli,’ ‘Toyonoka,’ ‘Sachinoka’ and ‘Benihoppe’ after 72 h of *C. gloeosporioides* infection. **(B)** Disease incidence on the leaves of the strawberry cultivars after 72 h of *C. gloeosporioides* infection. **(C)** Disease index values of the strawberry cultivars after 72 h of *C. gloeosporioides* infection. ^∗∗^*p* < 0.05.

In northern China, ‘Yanli,’ ‘Toyonoka,’ ‘Sachinoka’ and ‘Benihoppe’ are the main strawberry cultivars. The different cultivars responded differently to the pathogen *C. gloeosporioides.* We observed the leaves of ‘Yanli,’ ‘Toyonoka,’ ‘Sachinoka’ and ‘Benihoppe’ after 72 h of infection with *C. gloeosporioides*. Disease symptoms appeared in the strawberry cultivars (**Figure 1A**). The disease incidence and index values were determined at this time point, and statistical analysis was then performed (**Figures 1B,C**). The disease incidence and index values were the lowest for ‘Yanli’ and the highest for ‘Benihoppe’ among the four cultivars.

### Transcriptomes of Resistant and Susceptible Strawberry Cultivars Infected with *C. gloeosporioides*

To identify the genes that are responsive to *C. gloeosporioides* infection in strawberry and compare gene expression patterns between resistant and susceptible cultivars, six libraries were constructed from *Colletotrichum gloeosporioides*-infected leaf tissues and from non-vaccinated leaf tissues as a control. A total of 16.12 G and 16.05 G reads were sequenced from the ‘Yanli’ (R1, R2, and R3) and ‘Benihoppe’ (S1, S2, and S3) cultivars, respectively, and 206433226 clean reads were obtained from the six libraries (**Table 1**).

**Table 1:** Number of reads based on Illumina sequencing data in libraries.

Sample	Raw reads	Raw bases	Clean reads	Clean bases	Valid ratio (base)	Q30 (%)	GC content (%)
R1	37269064	5590359600	37269064	5590329804	99.99%	115.11%	46.50%
R2	36119336	5417900400	34113624	5108386065	94.28%	87.31%	47.00%
R3	34197908	5129686200	34197908	5129658786	99.99%	114.51%	45.50%
S1	37075374	5561306100	34599742	5180515674	93.15%	86.17%	46.00%
S2	36549070	5482360500	34631914	5186131672	94.59%	87.64%	47.00%
S3	33450420	5017563000	31620974	4735185696	94.37%	87.45%	46.00%

**FIGURE 2:**
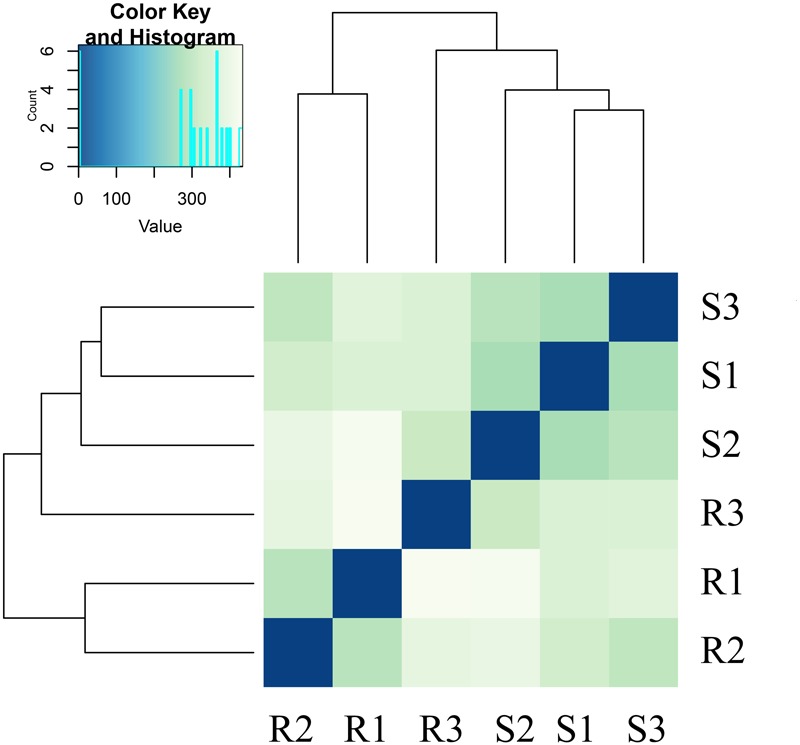
**Global changes in gene expression in the resistant strawberry cultivar ‘Yanli’ and the susceptible strawberry cultivar ‘Benihoppe’ during *C. gloeosporioides* infection.** Overall clustering of six samples using all normalized count data calculated by Cuffdiff. R1: 0-h-untreated ‘Yanli,’ R2: 72-h-untreated ‘Yanli,’ R3: 72-h-infected ‘Yanli’; S1: 0-h-untreated ‘Benihoppe,’ S2: 72-h-untreated ‘Benihoppe,’ S3: 72-h-infected ‘Benihoppe.’

The reads were compared with the octoploid strawberry genome FAN_r1.1.^6^ Gene expression levels in the RNA-seq analysis were measured as RPKM (**Supplementary Table S1**). The analyses revealed pairwise alterations of gene expression. R3 did not cluster with R1 and R2, and S3 did not cluster with S1 and S2 (**Figure 2**). The heat map, drawn from all gene count data from both genome references based on Euclidean distances, depicts the relationships of all of the transcriptomes.

The genes were annotated based on their similarity to known or putative sequences in web databases. Among the 230838 genes, 163033 (70.63%) had at least one significant match to a gene model according to the BLAST search (**Table 2**). The expressed genes were categorized according to GO and KEGG pathway analyses. All of the genes were queried against the Plant Resistance Gene database^7^ using BLASTp, and a total of 5227 putative R genes were identified. A list of the identified R genes is provided in **Supplementary Table S2**.

**Table 2:** Summary of strawberry gene annotations.

Category	Annotation number	Annotation ratio (%)
Nr_Annotation	163033	70.63
Swiss-Prot_Annotation	83140	36.02
KOG_Annotation	98812	42.81
R-Annotation	5227	2.26

### Transcriptomic Analysis of *C. gloeosporioides*-responsive Genes in Strawberry

**FIGURE 3:**
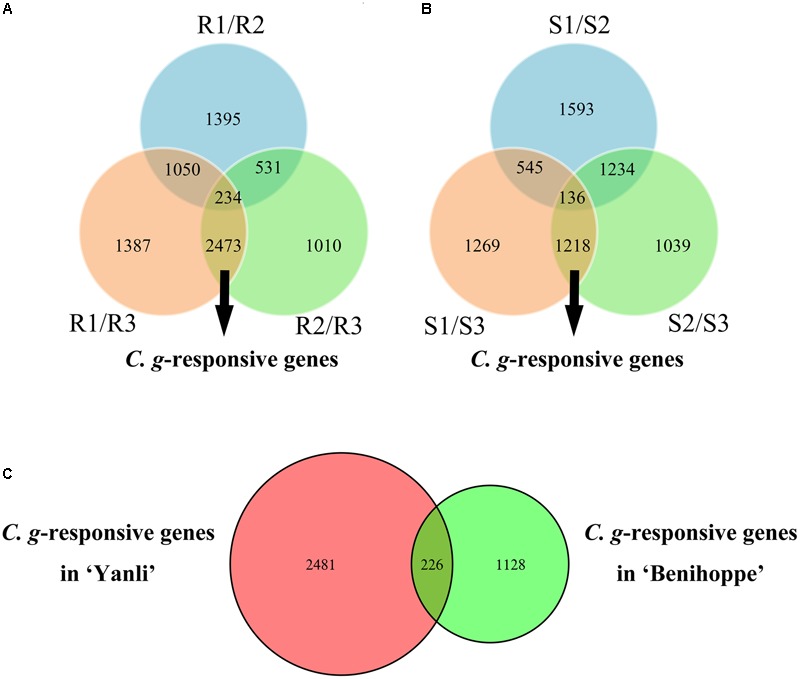
**Global changes in *C. gloeosporioides*-responsive genes in resistant and susceptible strawberry cultivars. (A)** The number of common and specific DEGs in ‘Yanli.’ **(B)** The number of common and specific DEGs in ‘Benihoppe.’ **(C)** The number of common and specific *C. gloeosporioides*-responsive genes in ‘Yanli’ and ‘Benihoppe.’

To identify the *C. gloeosporioides*-responsive genes in resistant and susceptible strawberry cultivars, R3 was compared with R1 and R2, with a union set of 2707 DEGs as *C. gloeosporioides*-responsive genes in ‘Yanli’ (**Figure 3A**), and S3 was compared with S1 and S2, with a union set of 1354 DEGs as *C. gloeosporioides*-responsive genes in ‘Benihoppe’ (**Figure 3B**). Then, the *C. gloeosporioides*-responsive genes were compared between ‘Yanli’ and ‘Benihoppe’ to reveal genes that were highly conserved or divergent in response to infection (**Figure 3C**). In total, we identified 2481 unigenes in ‘Yanli’ (**Supplementary Table S4**), 1128 unigenes in ‘Benihoppe’ (**Supplementary Table S5**), and 226 unigenes that intersected.

**FIGURE 4:**
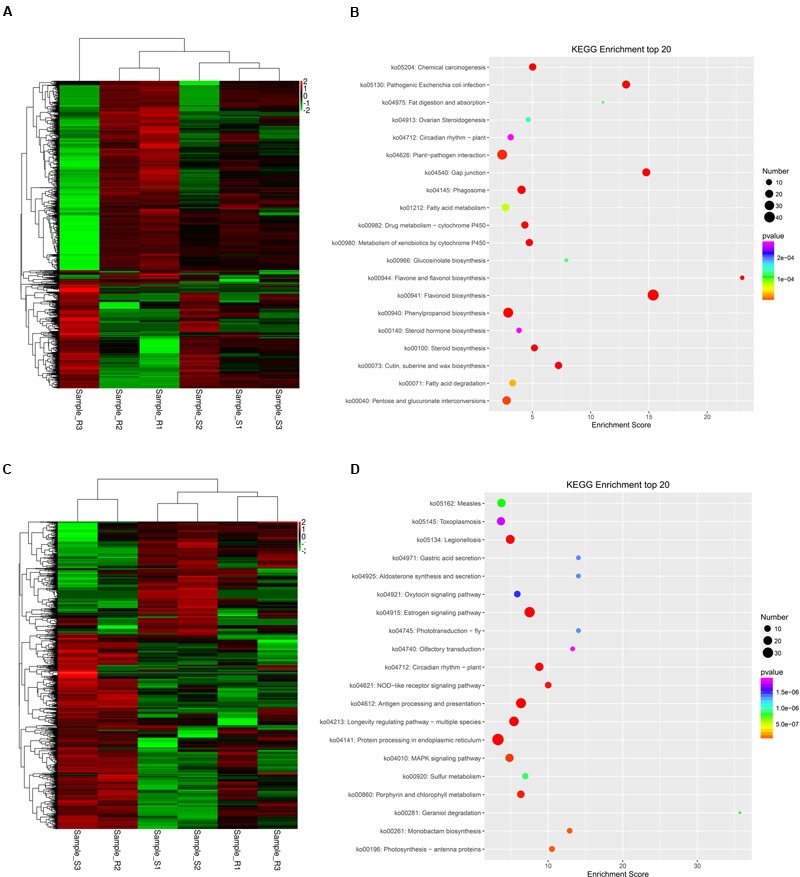
**Specific *C. gloeosporioides*-responsive genes in ‘Yanli’ or *‘*Benihoppe.*’* (A)** Expression patterns of *C. gloeosporioides*-responsive genes specific to ‘Yanli’ among the six pools. **(B)** KEGG enrichment analyses of *C. gloeosporioides*-responsive genes specific to ‘Yanli.’ **(C)** Expression patterns of *C. gloeosporioides*-responsive genes specific to ‘Benihoppe’ among the six pools. **(D)** KEGG enrichment analyses of *C. gloeosporioides*-responsive genes specific to *‘*Benihoppe.’

The expression patterns of these unigenes were analyzed in ‘Yanli’ and ‘Benihoppe’ during infection with *C. gloeosporioides* according to their FPKM values. The unigenes responsive to *C. gloeosporioides* infection in ‘Yanli’ but not responsive in ‘Benihoppe’ are shown in **Figure 4A**; those responsive in ‘Benihoppe’ but not ‘Yanli’ are shown in **Figure 4C**. To functionally classify these unigenes, we observed significant enrichment of these genes in multiple GO categories. The top GO terms with unigenes in ‘Yanli’ included cell wall organization process, flavonoid biosynthetic process, cutin biosynthetic process, response to biotic stimulus process and plant-type secondary cell wall biogenesis. The top KEGG pathways with unigenes in ‘Yanli’ included Flavonoid biosynthesis, Phenylpropanoid biosynthesis, Gap junction, Plant–pathogen interaction and Pathogenic *Escherichia coli* infection (**Figure 4B**). In ‘Benihoppe,’ 1128 unigenes were enriched in GO terms including response to heat, defense response to other organism, response to stress, and response to cold. The top KEGG pathways with unigenes in ‘Benihoppe’ included Protein processing in endoplasmic reticulum, MAPK signaling pathway, and Estrogen signaling pathway (**Figure 4D**).

**FIGURE 5:**
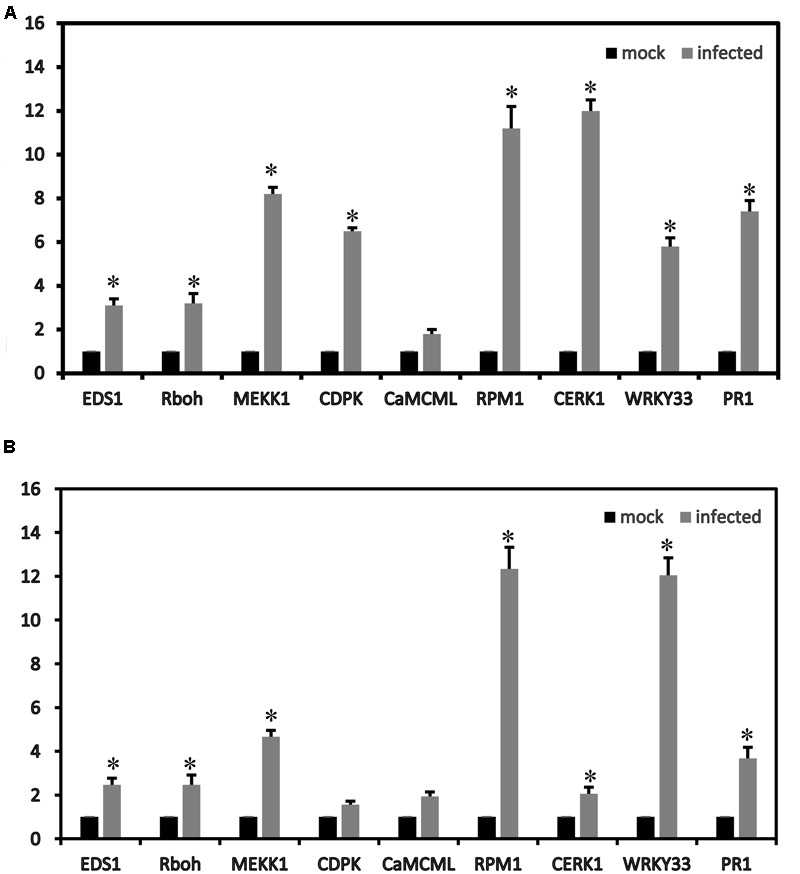
**Relative expression values according to the qRT-PCR analysis of the up-regulated strawberry genes in the plant–pathogen interaction pathways between mock and infected leaves at 72 h of ‘Yanli’ (A)** and ‘Benihoppe’ **(B)**. The qPCR results were generally in accordance with the gene expression profiles in the transcriptome data. ^∗^*p* < 0.1.

To confirm the RNA-seq results, some genes were validated using qRT-PCR. In the plant–pathogen interaction pathways, nine strawberry genes (*CERK1, EDS1, RPM1, CNGCs, CDPK, Rboh, CaMCML, WRKY33* and *PR1*) were up-regulated. The gene expression patterns (nine DEGs in the plant–pathogen interaction pathways) determined by qRT-PCR showed trends similar to those found in the RNA-seq data, which suggested that our transcriptome analysis was reliable (**Figure 5**).

## Discussion

This study was designed to identify strawberry genes related to defense against anthracnose. *C. gloeosporioides* is the primary infectious species in China. *C. gloeosporioides* typically causes petiole and stolon lesions and crown rot in strawberry and can also result in symptoms in the fruit (McInnes et al., 1992). ‘Yanli,’ ‘Toyonoka,’ ‘Sachinoka’ and ‘Benihoppe’ are the main strawberry cultivars in northern China. We conducted a disease assessment and found that disease symptoms appeared within 72 h, with ‘Yanli’ being a resistance accession and ‘Benihoppe’ being a susceptible accession. However, when a reference genome is available, the sequencing reads are aligned primarily by mapping onto the sequenced reference genome. The genome of *F. × ananassa* was firstly sequenced in 2013 (Hirakawa et al., 2013). We infected the ‘Yanli’ and ‘Benihoppe’ strawberry cultivars with *C. gloeosporioides* and compared their transcriptomes.

A total of 32 G of raw sequence data were generated via Illumina sequencing of strawberry leaves with or without *C. gloeosporioides* infection. In response to pathogen attacks, plants have evolved complex signaling and defense pathways. The transcriptomic analysis indicated that infection *C. gloeosporioides* elicited a wide range of responses. Many of the identified genes encode proteins with demonstrated roles in resistance and defense functions.

In plants, innate immunity is triggered via the response of PRRs to PAMPs (pathogen-associated molecular patterns), thereby providing the first line of inducible pathogen-associated molecular PTI. Chitin is one of the most common PAMPs in fungi and is highly conserved in this group (Boller and Felix, 2009; Chen and Ronald, 2011; Gust et al., 2012). CERK1 belongs to the PRR protein family. The stimulation of PRRs leads to PTI. These genes may be involved in PTI in strawberry against *C. gloeosporioides*.

The second class of receptors involves gene-for-gene-type interactions, which are used by the plant’s extracellular receptors to recognize pathogen virulence molecular effectors, thereby resulting in ETI. ETI is often initiated by a subset of R genes. The evolution of new plant disease-resistance genes is subject to strong selection pressure, and these genes play key roles in recognizing proteins expressed by specific pathogen-derived AVR genes and provide stable resistance for many generations (Ellis et al., 2000). In this study, 5227 R genes were identified in strawberry (**Supplementary Table S2**).

Plants can induce transcription factors to DNA sequences as a method of adjusting to biological and abiotic stresses (Atkinson and Urwin, 2012). Pathogen-response genes, such as WRKY, play crucial roles in plant responses. The WRKY family has been identified in the plant kingdom, and different WRKY families are found in higher plants (Ulker and Somssich, 2004). In strawberry, Casado-Díaz et al. (2006) found that the strawberry *Fawrky-1* gene may be associated with the concerted gene activation cascade that occurs within the mechanisms of plant defense. In this study, certain DEGs encoding WRKY exhibited different expression patterns, and we found that *WRKY33* transcripts were accumulated in response to *C. gloeosporioides* (**Figure 4**).

Plants can produce pathogenesis-related (PR) proteins in pathological-related environments. Various studies have suggested that PR genes play important roles in disease resistance. The expression level of PR1 was found to increase with pathogen infection (Palm and Medzhitov, 2009). PR1 expression increases during infection by *Meloidogyne incognita* in *Arabidopsis thaliana* (Hamamouch et al., 2011). The over-expression of CABPR1 in *Nicotiana tabacum* was found to be consistent with a significant increase in resistance following inoculation with pathogenic fungi (Sarowar et al., 2005). We found that PR1 expression was up-regulated in response to infection, thus supporting the SA pathway response.

In ‘Yanli,’ C. *gloeosporioides*-responsive unigenes were enriched in Plant–pathogen interaction pathway (**Figure 4B**). In this pathway, genes including CNGCs, CDPL, and Rboh were up-regulated. Calcium-mediated signaling was also activated because the ROS pathway is responsible for the hyper-sensitive response. All of the strawberry CNGC genes were up-regulated after infection, indicating that Ca^2+^ participates in signal transduction during early stage strawberry defense responses. Calcium is an essential second messenger in the signal transduction pathways that regulate plant responses to fungi (Zipfel, 2009). Ca^2+^ can activate RBOH protein, which is involved in the production of reactive oxygen species *in vitro* (Sagi and Fluhr, 2001). Our results indicated that RBOH was up-regulated (**Figure 4**), which is consistent with previous observations in wheat, cotton, cucumber, and *Brassica rapa* after infection by the fusarium wilt fungal pathogen (Dowd et al., 2004; Chen et al., 2015). This result indicates that higher ROS levels occurred in the strawberry leaves after infection.

Among ‘Yanli,’ ‘Toyonoka,’ ‘Sachinoka’ and ‘Benihoppe,’ ‘Yanli’ was resistant and ‘Benihoppe’ was susceptible (**Figure 1**). Intriguingly, the expression of most genes differed significantly between ‘Yanli’ and ‘Benihoppe.’ There were 4013 DEGs between R1 and S1, 3914 between R2 and S2, and 3895 between R3 and S3. The *C. gloeosporioides*-responsive genes also differed significantly (**Figure 3**). The expression of most genes differed significantly between ‘Yanli’ and ‘Benihoppe,’ indicating that ‘Yanli’ and ‘Benihoppe’ responded differently to the pathogen *C. gloeosporioides*. Furthermore, the *C. gloeosporioides*-responsive genes differed significantly and were enriched in different pathways, supporting the hypothesis that these cultivars may have different networks of defense mechanisms mediated by a diverse array of signaling molecules. More work is needed to understand the complex network of defense signaling pathways in strawberry. Our RNA-seq data provide molecular information relating to the defense response in strawberry.

## Conclusion

In conclusion, RNA-seq was utilized to reveal the transcriptomic response of the strawberry cultivars ‘Yanli’ and ‘Benihoppe’ inoculated with *C. gloeosporioides*, the causal agent of anthracnose. The DEGs were confirmed by qRT-PCR, which demonstrated the accuracy and reliability of the RNA-seq data. GO and KEGG enrichment analyses established that plant–pathogen interaction pathways play key roles in strawberry resistance to *C. gloeosporioides*. In strawberry, resistance to a variety of pathogens has been reported to be mostly polygenic and quantitatively inherited, making it difficult to associate molecular markers with disease-resistance genes. Our results further our understanding of the strawberry immune system and may facilitate future disease control through biotechnological and breeding strategies.

## Author Contributions

ZL, JF, and YM planned and organized the study. ZZ provided the plant material. FW and MC performed the *Colletotrichum gloeosporioides* culture, infection and RNA isolation and quality assessment. FZ performed qRT-PCR measurements. FW, FZ, and YM analyzed the data. FW, FZ, MC, and YM drafted the manuscript.

## Conflict of Interest Statement

The authors declare that the research was conducted in the absence of any commercial or financial relationships that could be construed as a potential conflict of interest.

## Footnotes

1 http://strawberry-garden.kazusa.or.jp//

2 http://www.ncbi.nlm.nih.gov

3 http://www.expasy.ch/sprot

4 http://prgdb.crg.eu/

5 http://bioconductor.org/packages/release/bioc/html/DESeq.html

6 http://strawberry-garden.kazusa.or.jp/

7 http://prgdb.crg.eu/

## Supplementary Material

TABLE S1 **Gene IDs and expression data**.

TABLE S2 **R genes**.

TABLE S3 **Primers used for qRT-PCR**.

TABLE S4 ***Colletotrichum gloeosporioides*-responsive genes specific to ‘Yanli’**.

TABLE S5 ***Colletotrichum gloeosporioides*-responsive genes specific to ‘Benihoppe’**.
